# The anti-MRSA efficacy of pyrrolomycin 5 by targeting membrane and in vivo therapeutic efficacy for MRSA-infected wound repair

**DOI:** 10.1007/s13659-026-00647-7

**Published:** 2026-11-09

**Authors:** Kaidi Qiu, Siran Li, Fangfang Duan, Yu Yang, Yunfei Yuan, Wei Gu, Xiaoyi Wei, Haibo Tan, Hongxin Liu

**Affiliations:** 1 School of Life and Health Technology, Dongguan University of Technology, Dongguan, 523808 China; 2 Key Laboratory of South China Agricultural Plant Molecular Analysis and Genetic Improvement, Guangdong Provincial Key Laboratory of Applied Botany, South China Botanical Garden, Chinese Academy of Sciences, Guangzhou, 510650 China; 3 State Key Laboratory of Natural and Biomimetic Drugs, School of Pharmaceutical Sciences, Peking University, Beijing, 100191 China; 4 State Key Laboratory of Functions and Applications of Medicinal Plants, Guizhou Medical University, Guiyang, 550014 China

**Keywords:** Pyrrolomycin 5, Methicillin-resistant *Staphylococcus aureus* (MRSA), Antibacterial mechanism, Membrane targeting

## Abstract

The emergence and spread of methicillin-resistant *Staphylococcus aureus* (MRSA) represent a critical global health threat, heightened by widespread antibiotic overuse during the COVID-19 pandemic. The urgent situation drives the critical appealing discover novel, efficient, and non-toxic anti-MRSA drugs. This study initiated with screening natural products from Lingnan’s characteristic medicinal plants and bacteria, leading to the identification of a pyrrolomycin-type compound, pyrrolomycin 5 (PM5). PM5 exhibited potent anti-MRSA activity with a minimum inhibitory concentration (MIC) of 0.78 μg/mL, comparable to that of vancomycin. It also demonstrated broad-spectrum efficacy against Gram-positive bacteria with a low propensity for resistance development. Mechanistic studies indicated that PM5 initially embeds into and depolarizes the bacterial membrane upon contact. As the concentration increases, PM5 accumulates on the membrane, ultimately compromising its integrity, causing rupture, and leading to bacterial cell death via leakage of cellular contents. The safety of PM5 was evaluated through in vitro cytotoxicity tests, and it was successfully applied in the treatment of skin infections in mice. In conclusion, PM5 represents a promising lead compound with a novel membrane-disrupting mechanism. This study provides valuable insights into its anti-MRSA mechanism and therapeutic efficacy. The findings offer a significant foundational framework for further development of pyrrolomycin-based antibiotics.

**Graphical abstract:**

**Figure:**
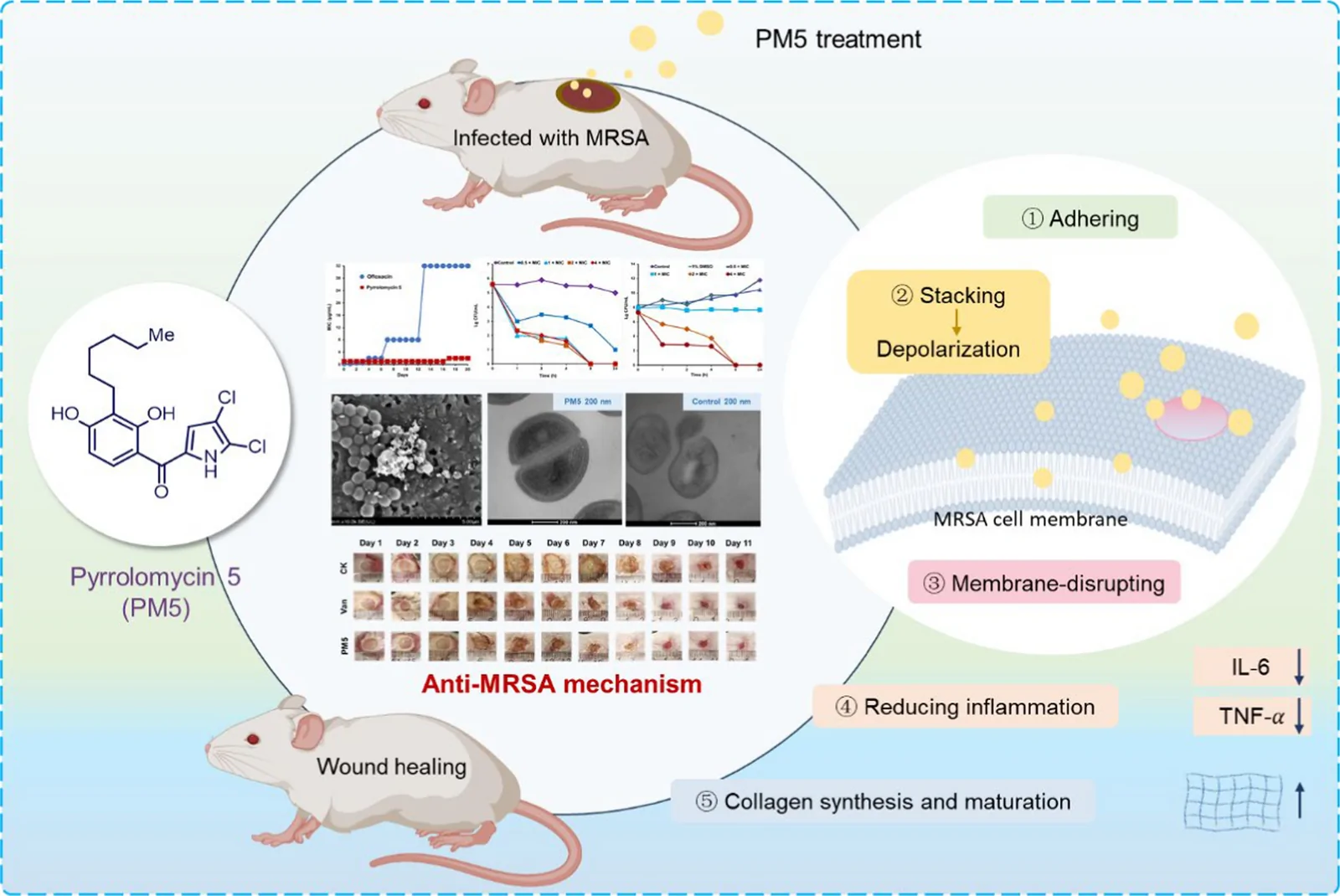

## Introduction

Bacterial infections have remained a persistent and major challenge to global public health, contributing significantly to worldwide disease burden and deaths [1]. Beginning with the discovery of penicillin in 1928, the advent of antibiotics ushered in a new era, providing an unprecedented weapon against bacterial infections [2]. Nevertheless, the inevitable emergence and subsequent spread of antimicrobial resistance, fueled by widespread misuse, foreshadow a concerning post-antibiotic future [3]. Although global efforts have encouraged more prudent antibiotic use, the COVID-19 pandemic has brought new challenges [4]. The initial overuse of antibiotics and their persistent misuse in treating COVID-19 in some regions have further intensified the global antimicrobial resistance crisis [5–7]. These troublesome situations highlight the urgent need to develop innovative antibacterial pharmaceutics to combat bacterial infections.

Among resistant pathogens, methicillin-resistant *Staphylococcus aureus* (MRSA) epitomizes the threat of "superbugs", posing a particularly formidable healthcare challenge [8]. Its high virulence, multidrug resistance, and mortality rates represent a major public health challenge, a situation further exacerbated by antibiotic practices during the pandemic [9]. Consequently, there is an urgent need to introduce novel antimicrobial molecules into clinical practice, coupled with the development of alternative strategies to combat antibiotic resistance. Historically, plants and bacteria have been continuingly served as an available reservoir to discovery novel therapeutic agents to combat bacterial infection, and the current strategies are still centered on the discovery of novel natural antibiotics and the chemical optimization of existing agents [10–12].

**Fig. 1:**
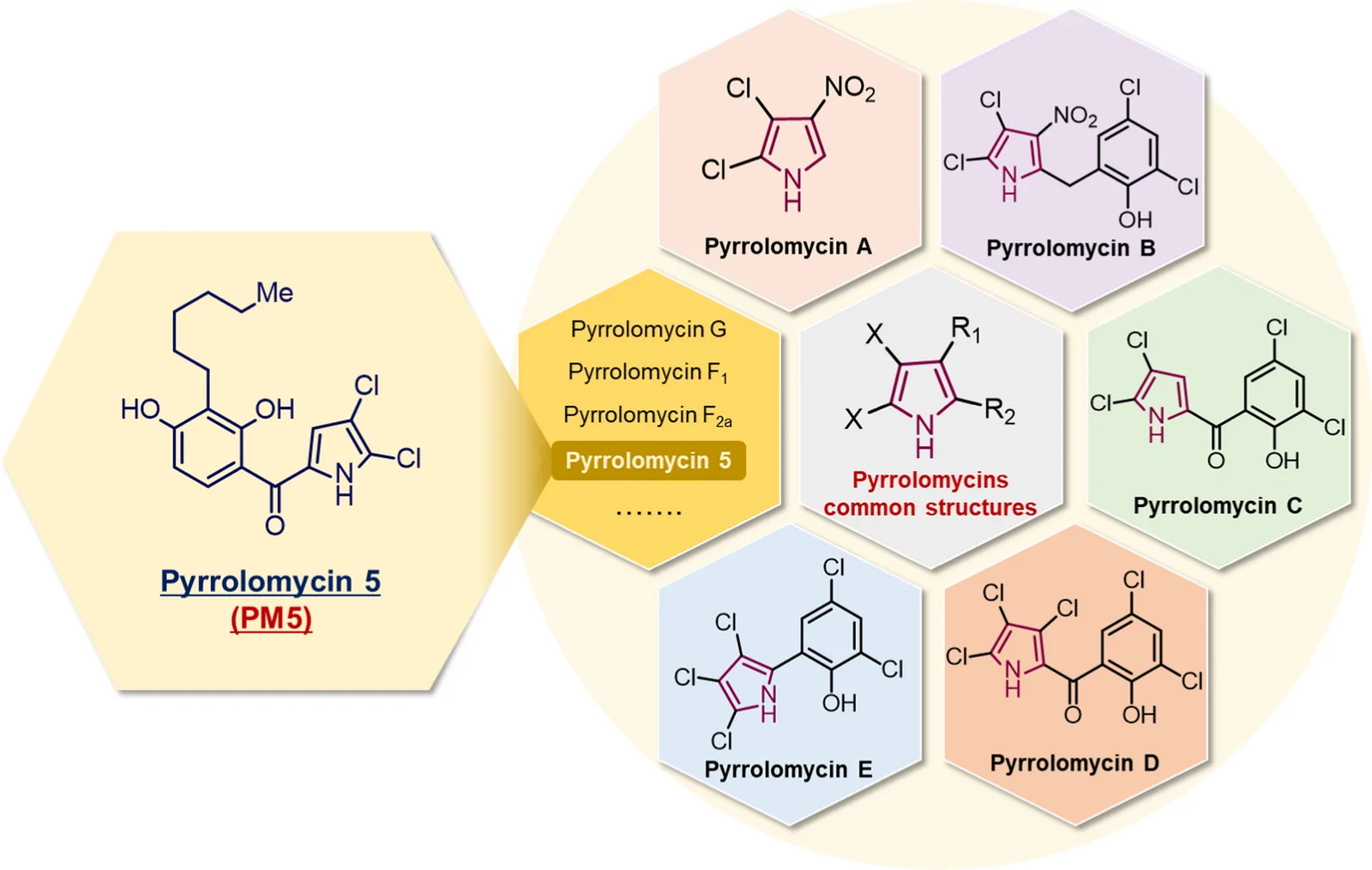
Representative natural pyrrolomycins and pyrrolomycin 5

Natural pyrrolomycins (PMs), polyhalogenated antibiotics derived from *Actinobacteria* and *Streptomyces*, are characterized by a highly chlorinated pyrrole ring bearing an intriguing aromatic phenyl substituent. Since the discovery of pyrrolnitrin in 1965 [13, 14], subsequent natural product research has successfully identified various PM derivatives demonstrating broad-spectrum antibacterial activity, potent efficacy against Gram-positive bacteria, and outstanding biofilm-inhibition capabilities [15–20]. Pyrrolomycins comprise a series of structurally fascinating and biologically meaningful natural products, including representative members such as dioxapyrrolomycin [18], as well as the series of chemically related derivatives designated as pyrrolomycins A-D as shown in Fig. 1, which show remarkable insecticidal, acaricidal, and antitumor activities. However, further pharmacological research towards the drug development of these compounds has been hindered by the focused preliminary activity assessments, with their mechanisms of action remaining elusive. Therefore, a deep understanding the mechanistic action of them could serve as an efficient way to reliably disclose their drug-like potential and thereby uncover novel chemically vulnerable bacterial targets.

Pyrrolomycin 5 (PM5), a novel PM derivative recently identified from *Streptomyces armeniacus*, exhibits potent antimicrobial activity comparable to that of vancomycin (Fig. 1) [21, 22]. However, studies on PM5 regarding its broader pharmacological potential and underlying its antibacterial mechanism are quite rare, primarily due to the challenges of obtaining sufficient quantities from natural sources. With the aforementioned considerations, it is crucial for the pharmaceutical community to deepen the understanding of this novel PM derivative and further explore its antibacterial potential as a promising antimicrobial lead compound for the treatment of drug-resistant Gram-positive bacterial infections. Therefore, this pharmacological study was carried out with the aim to assess its bactericidal efficiency against MRSA strain, elucidate its antibacterial mechanism, and evaluate the cytotoxicity assays, thus providing valuable insight into the practical strategies for combating MRSA. Furthermore, the in vivo efficacy of PM5 was successfully demonstrated in mice skin infection models, which deserves further exploration to be a potential lead for the development of new anti-MRSA agents.

## Materials and methods

### Reagents and chemicals

Resazurin was purchased from Sigma-Aldrich (USA). Vancomycin, polymyxin B, and melittin were sourced from Shanghai Aladdin Biochemical Technology. PBS was purchased from Wuhan Boster Biological Technology. DMSO was obtained from Shanghai Jizhi Biochemical Technology. The SYTOX green was purchased from Guangzhou Chengan Biotechnology. HEPES buffer solution was purchased from Shenzhen Yibaishun Technology. Brain Heart Infusion (BHI) broth and Mueller–Hinton (MH) broth were obtained from Guangdong Huankai Microbial Technology. All other chemicals were of analytical grade.

### Bacterial strains

*Staphylococcus aureus* (ATCC 6538, CMCC 26003), *Escherichia coli* (ATCC 8739, ATCC 8099, CMCC 44102), *Shigella dysenteriae* (CMCC 51252), *Candida albicans* (ATCC 10231) were obtained from the Guangdong Microbial Culture Collection Center (Guangzhou, China). Vancomycin-resistant *Enterococcus* (VRE, No. 151458137) and vancomycin-sensitive *Enterococcus* (VSE, No. 160119481) isolated from the patient's peritoneal cavity were donated by The First Affiliated Hospital, Sun Yat-sen University (Guangzhou, China). Clinical isolates of MRSA carrying *SCC-mec* (JCSC 3063, JCSC 4744, NCTC 10442, No.11646) were kindly provided by K. Hiramatsu and T. Ito from Juntendo University (Tokyo, Japan). A549, HeLa, and B2B cells were obtained from the Kunming Institute of Zoology, Chinese Academy of Sciences (Kunming, China). Bacteria were cultured in BHI or MH broth on a shaker bed at 200 rpm for 12 h at 37 °C, respectively. The bacteria suspension was centrifuged and washed with PBS, and its optical density at 600 nm was measured to determine the concentration of bacteria.

### Minimal inhibitory concentration (MIC) assay

The MICs of the synthesized compounds were determined using the microdilution method in 96-well microplates, following the CLSI standards [23]. Mix the bacterial log phase of approximately 10^6^ CFU/mL with resazurin solution (0.1 mg/mL) in a 2:3 ratio and dispense into a 96-well microplate. The stock solutions of compounds and vancomycin were prepared and added the culture medium for two-fold serial dilutions (100, 50, 25, 12.5, 6.25, 3.12, 1.56, 0.78 μg/mL). The tested bacterial were incubated at 37 °C for 8–12 h. Wells with 1% DMSO in the medium were used as the negative control and wells with vancomycin were used as the positive controls. MIC was calculated as the lowest concentration that inhibited visible growth of the bacteria.

### Multi-passage resistance assay

In this study, the 1/2 × MIC induction method was employed incorporating PM5 and ofloxacin as inducers. These inducers were co-cultured with MRSA at sublethal concentrations (1/2 × MIC) at 37 °C for consecutive intervals to simulate bacterial resistance development. Ofloxacin was used as the positive control. Using this method, we investigated the possibility of resistance development for the test compound, as deduced by alterations in MIC.

### Time-killing curve assay

The overnight culture of MRSA was adjusted in 0.85% NaCl to obtain a bacterial suspension with a concentration of 10^6^–10^7^ CFU/mL. The diluted bacterial suspension was treated with different concentrations (0, 0.5, 1, 2, and 4 × MIC) of PM5 and vancomycin, and incubated with shaking at 37 °C. At 0, 1, 2, 4, 8, and 24 h, the cell suspension was withdrawn and subjected to tenfold serial dilutions across a gradient of 10^0^–10^10^. Then, 100 μL of each diluted sample was spread onto MHA plates, which were inverted and incubated at 37 °C for 24 h. Colonies were counted to calculate the viable bacterial count (CFU/mL). Each experiment was carried out at least 3 times to obtain the mean value. Bactericidal activity was defined as a reduction of ≥ 3 log_10_ CFU/mL after 24 h of incubation compared with the blank control.

### Cytotoxicity

Cell proliferation and viability were assessed using the MTT [3-(4,5-dimethylthiazol-2-yl)-2,5-diphenyltetrazolium bromide] assay. Tumor cells were cultured in RPMI-1640 medium supplemented with 10% FBS and seeded into 96-well plates at a density of 10^4^ cells/well. After 24 h of adhesion, PM5 was added and incubated at 37 °C for 24 h. Subsequently, MTT was added to each well, followed by incubation at 37 °C for 4 h. The absorbance was measured at 490 nm, and the inhibition rate was calculated based on the absorbance values. The IC_50_ value was determined using SPSS 22.0 (IBM Inc., Chicago, IL, USA). Each experiment was performed in triplicate and independently repeated three times.

### Membrane permeability of MRSA

The integrity of the inner membrane of MRSA treated by PM5 was investigated by 3,3′-dipropylthiadicarbocyanine iodide (DiSC3(5)). The DiSC3(5) assay was conducted following the instruction of Sautrey et al. with minor modifications [24]. In brief, MRSA was centrifuged and diluted with 5 mM HEPES (containing 5 mM glucose) till OD_600_ of 0.1. 0.4 µM DiSC3(5) was added into the prepared bacteria solution and incubated at 37 °C with shaking at 200 rpm for 20 min in the dark. KCl solution was added with the final concentration of 100 mM. Fluorescence units were measured immediately at the excitation wavelength of 660 nm and emission wavelength of 675 nm for 10–20 min at 1 s intervals. Triple experiments were conducted with three individual clones of MRSA.

### Morphological assay of bacteria under cryo-scanning electron microscopy (Cryo-SEM)

The SEM was used to analyze the morphological changes of MRSA. MRSA was treated with PM5 (4 × MIC) at 37 °C for 4 h. The cells were then washed 3 times by centrifugation with 0.1 M PBS (pH 7.2) and resuspended in sterile ultrapure water. Bacterial suspension was spread on a dedicated filter membrane on the sample stage to absorb moisture, and fix the bacterial samples for electron microscopy observation and photography.

### Observation of bacterial structures under transmission electron microscope (TEM)

MRSA was diluted with PBS and combined with PM5. Following incubation at 37 °C for 4 h, the cells underwent centrifugation at 4000 rpm for 5 min and were subsequently washed twice with PBS. After overnight fixation with 2% glutaraldehyde at 4 °C, the cell pellet was collected and washed 6 times with PBS. Then, 1% osmium tetroxide was added to incubate for 2 h. Following three washes with PBS, the samples were dehydrated through a graded ethanol series, freeze-dried, stained, and subsequently observed and photographed under TEM.

### Observation of bacterial membranes under laser scanning confocal microscope (LSCM)

Integrity in membrane of MRSA cells treated with PM5 was tested by fluorescent-based cell live/dead under confocal laser scanning microscopy. MRSA was washed 3 times by centrifugation with HEPES buffer, and the bacterial suspension was adjusted to a turbidity corresponding to 1 × 10^8^ CFU/mL. Under light-protected conditions, 3 μM SYTOX GREEN, 50 nM DiSC3(5), and 1.5 μM PI were mixed with PM5 at concentrations of 0, 0.5, 1, 2, and 4 × MIC and co-incubated at 37 °C for 20 min. Bacterial states were then imaged using LSCM.

### Murine skin infection model with MRSA

The animal experiments were performed under a protocol approved by the Institutional Animal Care and Use Committee of the Guangdong Quality Supervision and Testing Station for Medical and Health Care Appliances (Approval No. K2024-01–183-262). 32 seven-week-old female ICR mice weighing 17–20 g were housed in Guangdong Quality Supervision and Testing Station for Medical and Health Care Appliances (license No. SYXK (Yue) 2023–0183 for Use of Experimental Animals). All animals were caged under standard environmental conditions, with ambient temperature regulated at 21–25 °C, relative humidity at 50%-60%, ad libitum access to food and water in cages on standard light: dark (12:12 h) cycles.

The ICR mice were randomly allocated to infected control, vancomycin (VAN, positive drug) and PM5 groups (n = 6 per group). After anesthesia, 1 cm diameter full-thickness dorsal wounds were created on shaved skin every mice, and 10 μL of MRSA suspension (1 × 10^8^ CFU/mL) was inoculated to establish infection models. The vancomycin and PM5 groups received daily topical administration of the corresponding agents, while the infected control group was treated with saline, and wound areas were measured at regular intervals. Wound areas were measured periodically, and tissues were collected for histopathological (H&E and Masson staining) and immunohistochemical (IL-6 and TNF-*α*) examinations.

### Statistical analysis

All experimental data are shown as means ± SEM, and statistically analyzed by SPSS 22.0 (IBM Inc., Chicago, IL, USA). The statistical significance was calculated by one-way ANOVA and differences were considered to be significant at *p* < 0.05. And GraphPad Prism 7 was applied for graphical plotting and analysis.

## Results and discussion

### Potent broad-spectrum antibacterial activity

To investigate whether PM5 exhibits broad-spectrum bacteriostatic and bactericidal effects, the in vitro antibacterial activity against a panel of 12 bacterial strains was evaluated by using the broth microdilution method. The tested strains included Gram-positive bacteria (MRSA NCTC 10442, MRSA JCSC 4744, MRSA 11646, MRSA JCSC 3063, *S. aureus* ATCC 6538, *S. aureus* CMCC 26003, VRE 151458137, VSE 160119481) and Gram-negative bacteria (*E. coli* ATCC 8739, *E. coli* CMCC 44102, *E. coli* ATCC 8099, *S. dysenteriae* CMCC 51252). The reference positive compounds were the commonly used clinical antibiotics vancomycin (VAN) and polymyxin (POL). The MIC data for the tested compound PM5 are shown in Table 1. As a result, PM5 effectively inhibits and kills the MRSA cells, demonstrating superior antibacterial activity compared to the first-line clinical anti-MRSA drug vancomycin. PM5 exhibited a MIC of 0.78 μg/mL against all tested Gram-positive strains, whereas its antibacterial activity against Gram-negative strains was weak, with MIC values of 100 μg/mL observed only against one *E. coli* strain and *S. dysenteriae*.

**Table 1:** Antibacterial activity of PM5 against susceptible bacteria

Bacteria	G^+^/G^−^	MIC (μg/mL)		
		PM5^a^	VAN^b^	POL^c^
MRSA NCTC 10442	G^+^	0.78	1.25	–
MRSA JCSC 4744	G^+^	0.78	1.25	–
MRSA No.11646	G^+^	0.78	0.62	–
MRSA JCSC 3063	G^+^	0.78	0.62	–
*S. aureus* ATCC 6538	G^+^	0.78	0.62	–
*S. aureus* CMCC 26003	G^+^	0.78	1.25	–
VRE No.151458137	G^+^	0.78	> 10	–
VSE No.160119481	G^+^	0.78	0.31	–
*E. coli* ATCC 8739	G^−^	100	–	2.5
*E. coli* CMCC 44102	G^−^	> 100	–	2.5
*E. coli* ATCC 8099	G^−^	> 100	–	2.5
*S. dysenteriae* CMCC 51252	G^−^	100	–	2.5

^a^PM5 (pyrrolomycin 5); ^b^VAN (vancomycin); ^c^POL (polymyxin)

Based on these results, PM5 demonstrates broad-spectrum antibacterial activity against Gram-positive bacteria, but it is largely ineffective against Gram-negative bacteria, indicating its potential to be developed as a selective lead compound toward innovative antibacterial agent. Moreover, PM5 showed notable and stable antibacterial activity against both susceptible and MRSA stains, suggesting that the existing MRSA resistance mechanisms do not obviously affect its antibacterial efficacy. These results collectively implies that the potential mechanistic target of PM5 may be different from the resistance targets of MRSA for the common clinical drugs. Therefore, the antibacterial action mode and mechanism of PM5 are likely novel and unlike those of vancomycin, and it does not involve inhibition of peptidoglycan synthesis in the cell wall [25, 26].

### Evaluation of potential resistance development

Evaluating the propensity of a novel antibacterial agent to drive resistance in MRSA is a fundamental aspect of its further drug development and an efficient methodology to highlight its potential antibacterial mechanism and reveal the possible pharmacological target. The bacterial resistance usually occurs through the necessary selective environmental pressure, because the drug eliminates susceptible bacteria, allowing pre-existing resistant mutants or subpopulations to survive and proliferate. The subsequent propagation of these resistant clones increases their prevalence within the population. Clinically, a fourfold or greater increase in the MIC against the same strain confirms the development of bacterial resistance [27].

**Fig. 2:**
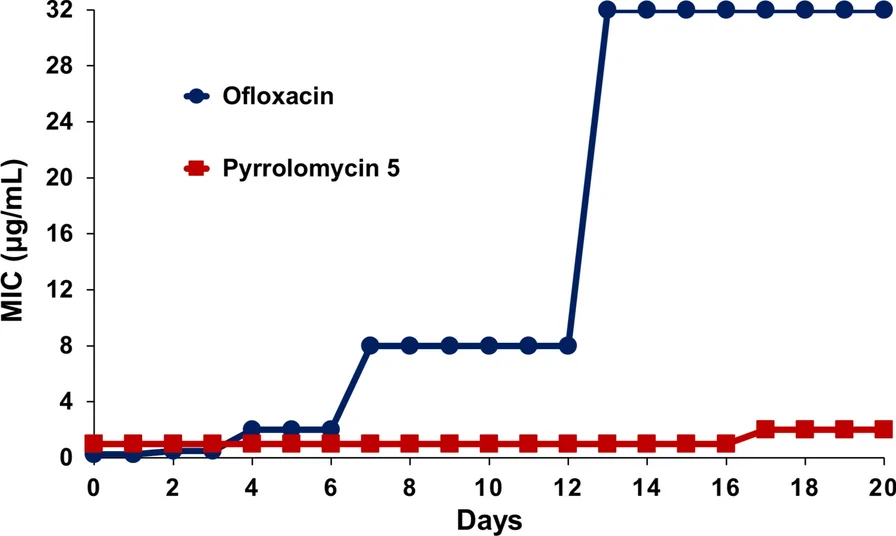
Bacterial resistance of PM5 against MRSA JCSC 3063 over times with ofloxacin as a positive control. Bacteria were passaged daily in the presence of increasing sub-inhibitory concentrations (0.5 × MIC) of PM5 or ofloxacin for 20 days. Data shown are absolute MIC values

To evaluate the antibacterial potential of PM5 to avoid endogenous mutational resistance in MRSA, the long-term passaging experiments with the treatment of sub-inhibitory concentrations of PM5 for MRSA cells were performed, and the conclusive results were comprehensively compiled as shown in Fig. 2. MRSA JCSC 3063 is a typical MRSA reference strain and is often used in membrane targeting and resistance development studies [28]. Ofloxacin, a fluoroquinolone with a known propensity to induce resistance [29], served as a control, its MIC increased from 0.25 to 0.5 μg/mL by the second passage and escalated to 32 μg/mL by the 13th passage, which was significantly resulted in a 128-fold rise from the initial MIC, demonstrating notable bacterial resistance. In contrast, the MIC of PM5 remained stable from passages 1 to 16, showing only a single two-fold increase (to 2 μg/mL) by the 17th passage. These intriguing findings suggest that PM5 showed a significantly lower tendency to induce resistance in MRSA as compared to ofloxacin.

### Bactericidal time-kill kinetics and growth-inhibiting curves

**Fig. 3:**
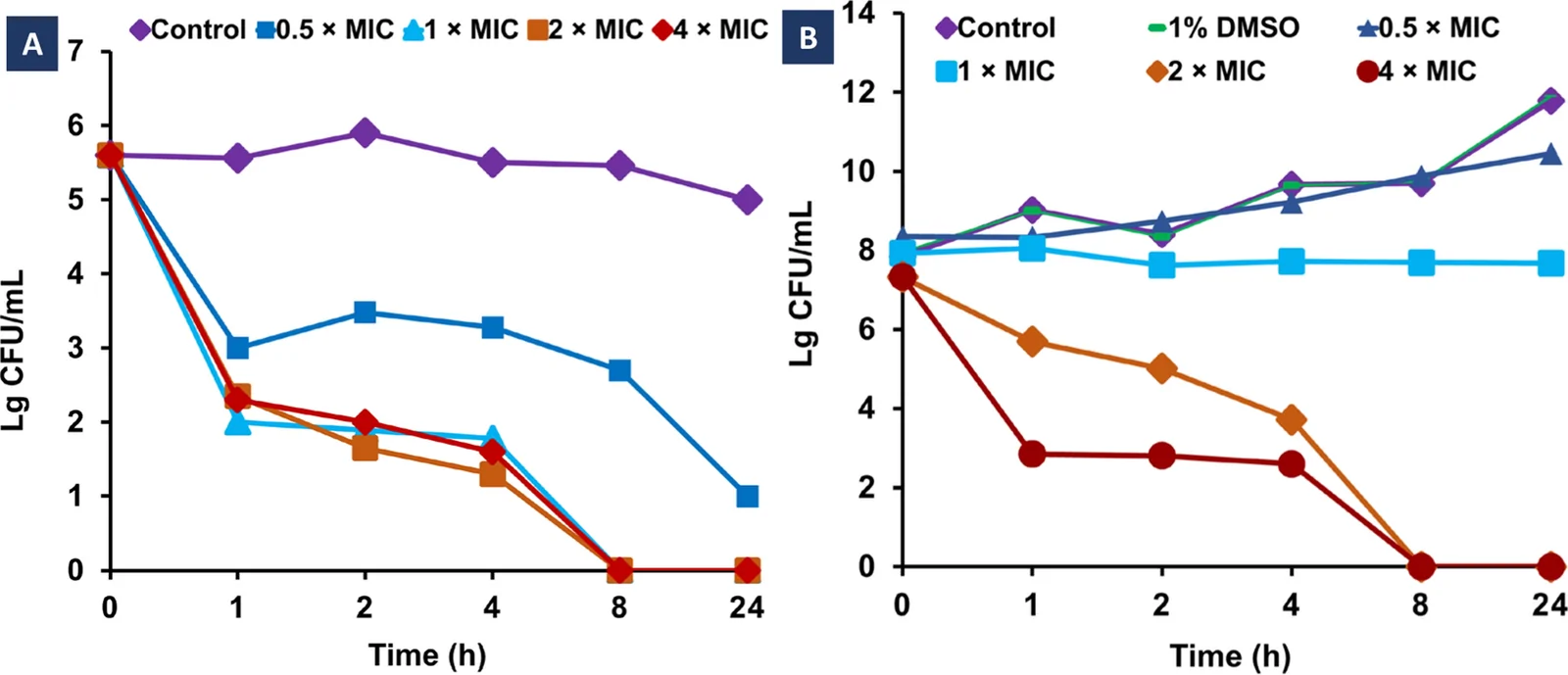
Bactericidal and growth‑inhibitory activities of PM5 against MRSA JCSC 3063. **A** Time‑kill kinetics. MRSA was diluted in sterile ultrapure water to 10^6^ CFU/mL. The bacterial suspension was then exposed to PM5 at 0.5 × , 1 × , 2 × , and 4 × MIC (MIC = 0.78 μg/mL). Sterile ultrapure water was used as the negative control. **B** Growth‑inhibitory curves. MRSA was diluted in MHB to 10^6^ CFU/mL. The bacterial suspension was then exposed to PM5 at 0.5 × , 1 × , 2 × , and 4 × MIC (MIC = 0.78 μg/mL). MHB was used as the negative control

Using a multi-time-point sampling approach, the in vitro dynamic bacteriostatic and bactericidal effects of PM5 against MRSA were assessed, including time-kill curves and growth inhibition curves, with results presented in Fig. 3 A and B. The time-kill curve results showed that the bacterial count in the control group remained stable within the range of 10^5^–10^6^ CFU/mL, effectively ruling out interference from variations in bacterial growth status. At 1 h after drug administration, the colony counts in all treatment groups were lower than those in the control group, with bactericidal rates exceeding 99.9%. Between 2 and 4 h, the number of surviving MRSA in the 0.5 × MIC group was higher than that in groups treated with higher concentrations. By 8 h, complete eradication of MRSA was observed in the 1 × , 2 × , and 4 × MIC groups, whereas a small number of bacteria remained in the 0.5 × MIC group even after 24 h of treatment. These findings are consistent with the MIC measurements of PM5, further confirming the accuracy of the antibacterial activity results described above.

The growth inhibition curve results showed that the bacterial count in the control group started at approximately 10^8^ CFU/mL and gradually increased, reaching about 10^12^ CFU/mL by 24 h. The trend observed in the 0.5 × MIC group closely resembled that of the control group. In contrast, the 1 × MIC group maintained MRSA levels near 10^8^ CFU/mL. Both the 2 × and 4 × MIC groups exhibited bactericidal effects at 1 h, with a 99% kill rate for the 2 × MIC group and 99.9999% for the 4 × MIC group. By 8 h, complete eradication of MRSA was achieved in both groups. These results confirm that PM5 exerts a rapid and potent inhibitory effect on the growth and proliferation of MRSA. Its bactericidal activity is both time-dependent and concentration-dependent, demonstrating favorable dynamic antibacterial efficacy.

### PM5 induces membrane depolarization and disruption of MRSA

The bacterial cell membrane depolarization refers to a reduction in membrane polarization or a shift of the resting potential toward less negative value inside the cell. This typical physiological process involves interactions between drugs and ion channels embedded in the cell membrane [30]. DiSC3(5), a carbocyanine dye with short alkyl tails, can accurately detect changes in transmembrane potential or membrane structure induced by membrane-active agents. It specifically accumulates within the phospholipid bilayer of live cells, leading to self-quenching of the dye [31]. When membrane-active compounds cause depolarization and loss of membrane potential, DiSC3(5) is released into the solution, resulting in a measurable increase in fluorescence. Complementary assays for membrane integrity employ dyes such as SYTOX GREEN and propidium iodide (PI) [32,33]. Both are nucleic acid stains that are normally excluded by intact, healthy cell membranes. When the cytoplasmic membrane is compromised, these dyes enter the cell, bind tightly to nucleic acids, and exhibit a substantial increase in fluorescence, thereby serving as indicators of cell death or membrane damage.

**Fig. 4:**
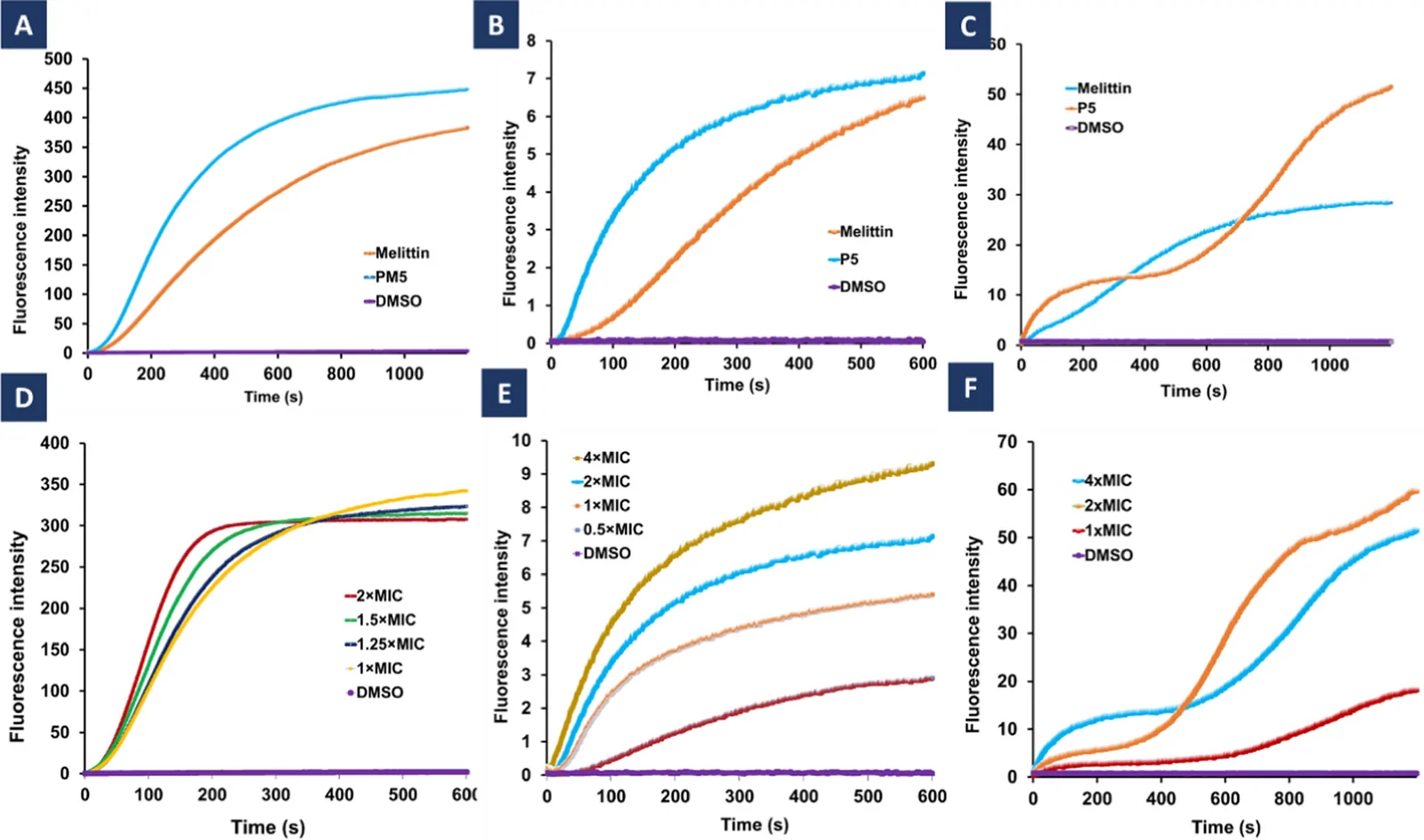
PM5 disrupts the cytoplasmic membrane of MRSA JCSC 3063 by inducing depolarization (DiSC_3_(5)) and increasing permeability (SYTOX Green and PI). **A**–**C** Representative fluorescence curves at selected concentrations of PM5 compared with the positive control melittin at the same concentration and negative control (1% DMSO). **D–F** Concentration‑dependent effects of PM5 on membrane permeability (**D**, **E**) and depolarization (**F**)

Based on the outstanding capacity of the aforementioned membrane fluorescence probes, the effect of PM5 on MRSA cytoplasmic membrane was evaluated using these three fluorescent dyes to measure membrane depolarization and permeability. Melittin was used as the positive control [34]. As shown in Fig. 4A–F, the addition of DiSC3(5) revealed a stable fluorescence curve in the control group, whereas the PM5-treated group induced two distinct, rapid increases in fluorescence, one immediately after treatment and another at approximately 500 s, indicating that PM5 can effectively cause MRSA membrane depolarization in a time-dependent manner. Furthermore, the immediate increase in fluorescence upon PM5 addition for both SYTOX GREEN and propidium iodide (PI) confirmed its robust ability to disrupt MRSA membrane integrity. Notably, PM5 exhibited stronger membrane-depolarizing and disruptive effects than the reference compound melittin at the same concentration, suggesting that PM5 is a potent membrane-targeted antibacterial agent.

**Fig. 5:**
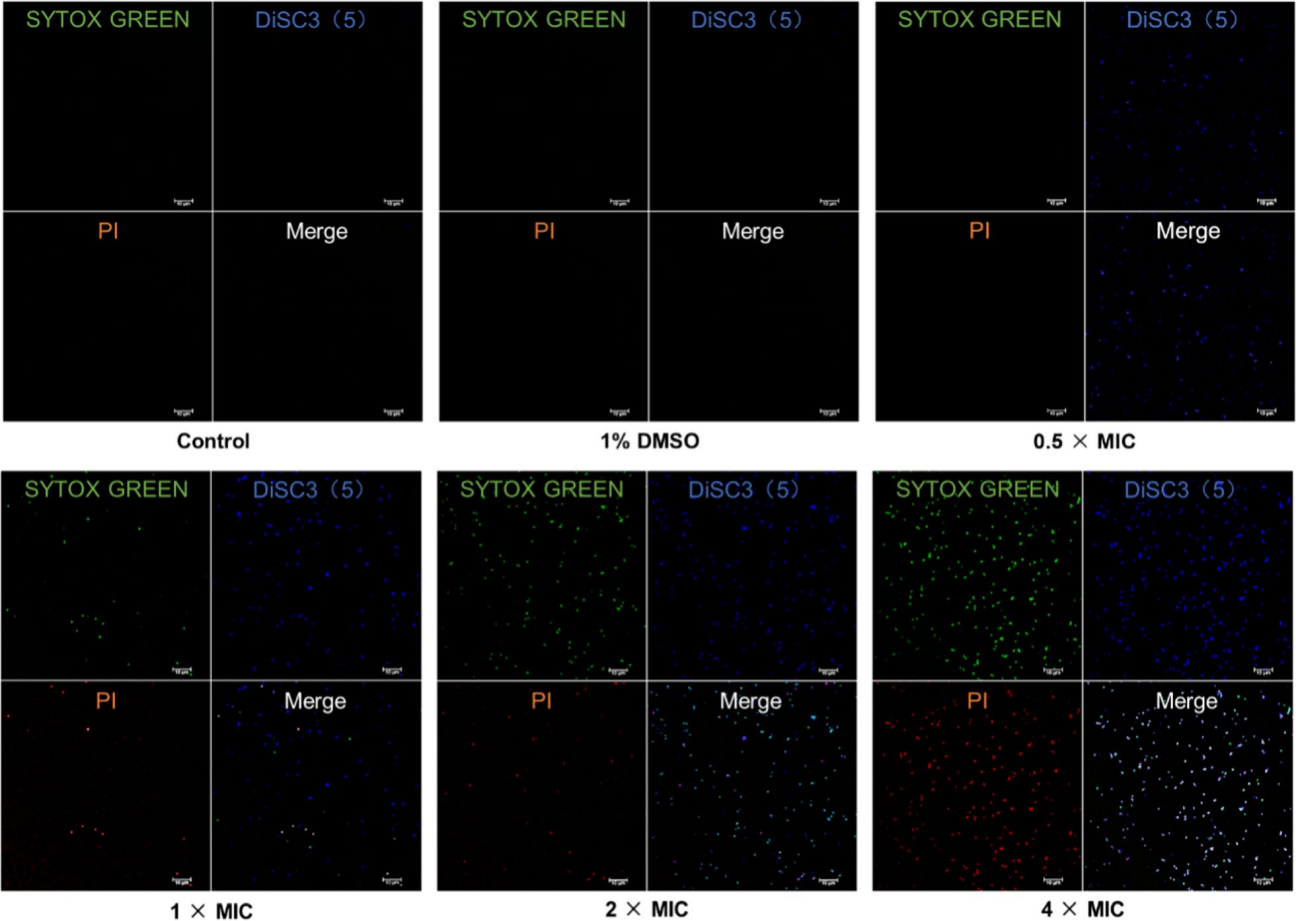
Confocal laser scanning microscopy (CLSM) images of MRSA JCSC 3063 after treatment with PM5 (0.5, 1, 2, 4 × MIC) and negative control (H_2_O and 1% DMSO)

To further confirm its outstanding membrane-disrupting efficacy, confocal microscopy was used to investigate PM5′ effects on MRSA membrane integrity and depolarization. As expected, PM5 efficiently induced the faint blue fluorescence from DiSC3(5) at a low concentration (0.5 μg/mL), while no obvious fluorescence induced by SYTOX GREEN or PI probe was observed (Fig. 5). These informative results suggested that only membrane depolarization occurred at this stage. At higher drug concentrations (> 1 μg/mL), PM5 could disrupt MRSA membrane integrity, as evidenced by SYTOX GREEN and PI fluorescence probes, whose fluorescence intensity were proportional to the drug concentration. These fluorescence experiments towards the membrane disruption successfully indicated that the PM5 exerted a concentration-dependent antibacterial activity, corroborating the results from membrane permeability and depolarization assays and further elucidating its potential mechanism of action.

Based on the aforementioned experimental results, the MRSA cytoplasmic membrane is identified as a key target of PM5, which exhibits stronger membrane disruption and depolarization effects than the positive control melittin [34]. The possible mechanism may involve an initial insertion of PM5 into the cell membrane, causing significant MRSA cell membrane depolarization. As the targeted compound PM5 accumulates on the MRSA membrane at higher concentrations, it directly disrupt the MRSA membrane integrity, rapidly leading to cell rupture and leakage of cellular contents, thus giving rise to the ultimate bacterial death.

### Microscopic and ultrastructural changes to MRSA caused by PM5

**Fig. 6:**
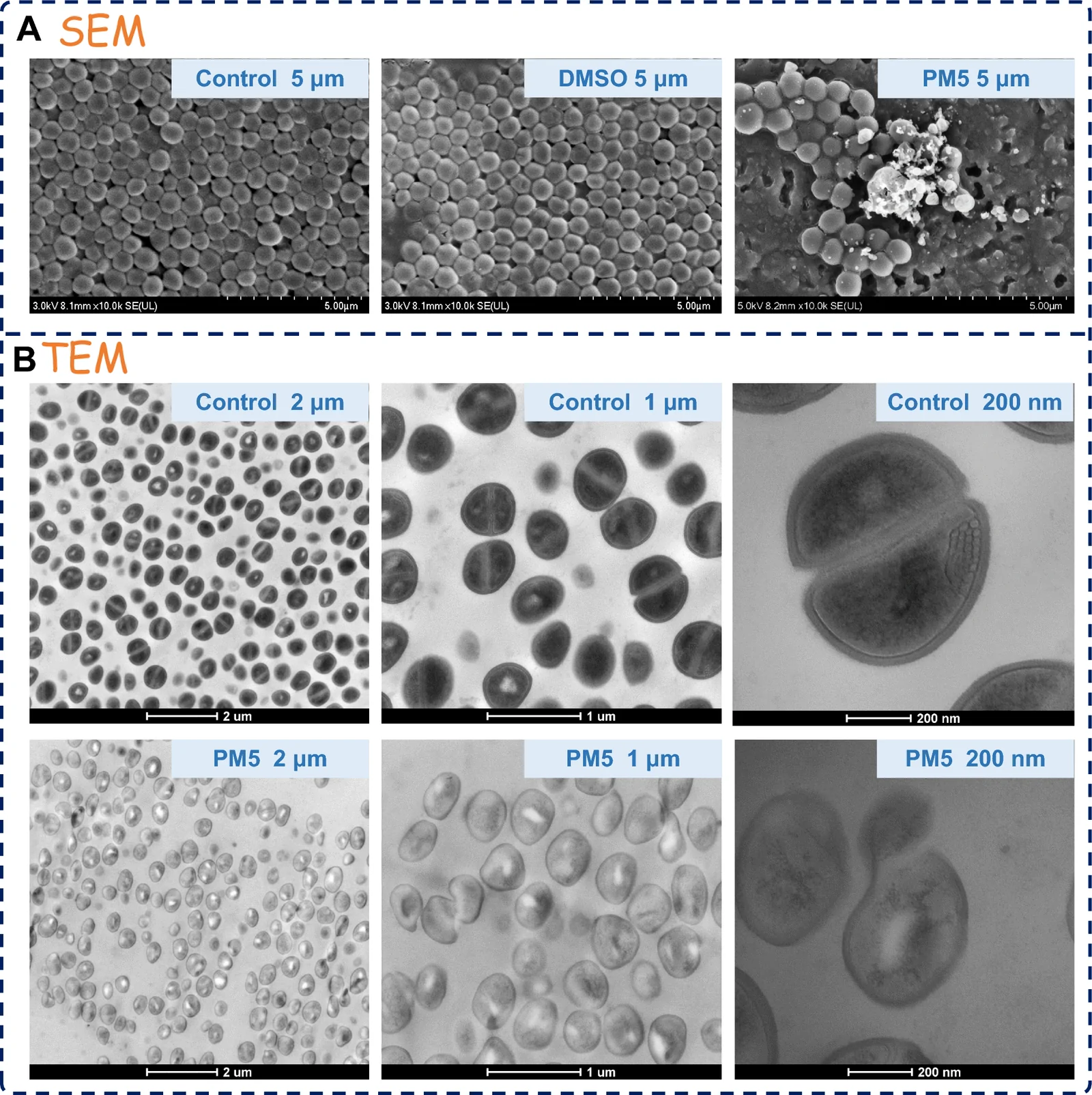
Microscopic and ultrastructural changes to MRSA caused by PM5. **A** SEM of MRSA JCSC 3063 treated with PM5; **B** TEM of MRSA JCSC 3063 treated with PM5

Subsequently, the morphology of MRSA cells treated with PM5 was examined using scanning electron microscopy (SEM) and transmission electron microscopy (TEM). As shown in Fig. 6, the SEM imaging revealed that the number of MRSA bacteria was significantly reduced in the PM5-treated group (4 × MIC), and many MRSA cells displayed ruptured cell structures (Fig. 6A and B). Specifically, the surface of MRSA exhibited numerous linear cracks, accompanied by cytoplasmic leakage and the formation of intracellular cavities, ultimately leading to bacterial death. Therefore, these conclusive ultrastructural results for the drug-treated MRSA cells provide direct evidence that PM5 could efficiently damage the cellular structure of MRSA to display its potent bactericidal efficacy.

Notably, TEM experiments were also conducted to further demonstrate the membrane-disrupting capacity of PM5. As shown in Fig. 6, normal MRSA bacteria in the control group maintained intact morphology, with clearly distinguishable cell walls and membranes, darker cell wall staining, and visible normal binary fission (Fig. 6A). In contrast, bacteria treated with PM5 (4 × MIC) showed lighter internal staining, blurred membrane boundaries, and ruptured cell structures, with extensive leakage of cellular contents leading to MRSA death (Figs. 6B). Therefore, PM5 likely exerts its bactericidal effect by targeting the MRSA cell membrane and causing bacterial rupture as a potent antibacterial agent, which further corroborates the aforementioned experimental conclusions.

### Cytotoxicity of PM5

To determine the in vitro cytotoxicity of PM5, this study employed the MTT assay to investigate the effects of PM5 on the viability of human non-small cell lung cancer cells (A549), human cervical cancer cells (HeLa), murine macrophage cells (RAW264.7), and human normal lung epithelial cells (B2B) (Table 2), with doxorubicin (ADM) serving as a positive control. MTT results showed that the IC_50_ value of PM5 was 7.30 μg/mL for A549 cells, 6.95 μg/mL for HeLa cells, 17.19 μg/mL for RAW264.7 cells, and 11.28 μg/mL for B2B cells. Based on the selectivity index (SI) [35], the half-maximal inhibitory concentration (IC_50_) of PM5 against cancer cells A549, HeLa, and RAW264.7 was more than eightfold higher than its minimum inhibitory concentration (MIC = 0.78 μg/mL) against MRSA. Simultaneously, the IC_50_ of this compound against normal cells B2B was more than 14-fold higher than its MIC against MRSA. This indicates that at 0.78 μg/mL, PM5 effectively inhibits MRSA growth while exhibiting relatively low toxicity to normal B2B cells and potentially demonstrating selective killing activity against tumor cells. Based on these findings, PM5 shows significant potential as an antimicrobial drug candidate, particularly for treating MRSA infections, warranting further in-depth research and development.

**Table 2:** In vitro cytotoxic activity of PM5

	IC_50_ (μg/mL)			
	A549	Hela	RAW264.7	B2B
PM5	7.30 ± 0.60	6.95 ± 0.75	17.19 ± 0.51	11.28 ± 0.04
ADM	1.16 ± 0.59	0.30 ± 0.35	0.96 ± 0.31	0.86 ± 0.71

### In vivo antibacterial effect

**Fig. 7:**
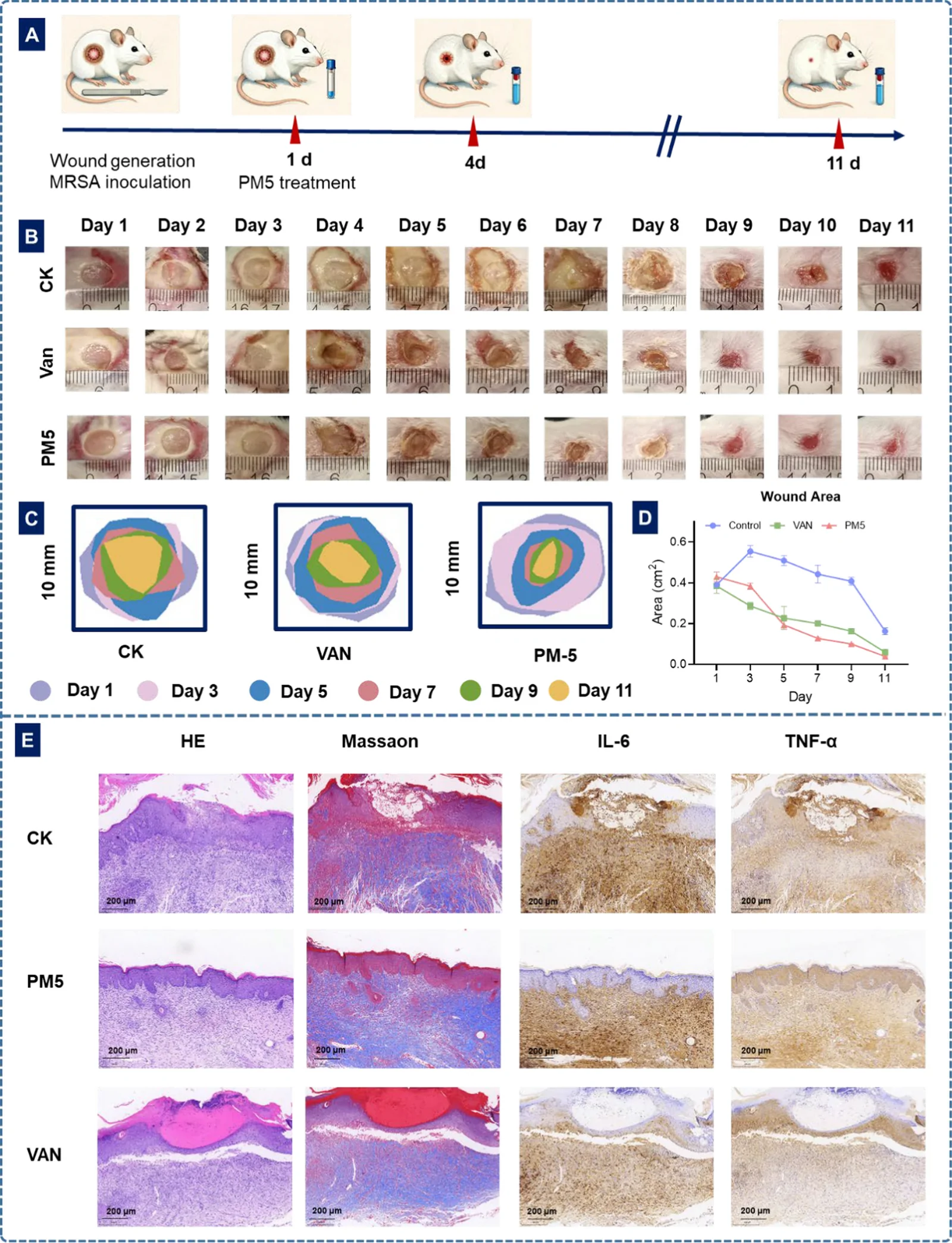
In vivo antibacterial assay of PM5. **A** Schematic of the experimental protocol for the mouse MRSA-infected model. **B**–**C** Photographs and corresponding wound area of the MRSA-infected wounds in PM5-treated groups on different days. **D** Repair diagram of mouse wound area. **E** Hematoxylin and eosin (H&E), Masson, and immunohistochemical staining of MRSA-infected wound tissues in CK, PM5, and VAN groups

The good biocompatibility of PM5 stimulates us to further construct MRSA-infected wound model in mice to evaluate its therapeutic potential. As shown in Fig. 7, the infected mice were randomly categorized into three groups, and the capacity of compound PM5 to mend full-thickness skin wounds was evaluated in comparison with the vancomycin (VAN) treatment group and the infected control (CK) group. Representative photographic documentations of full-layer wounds from the compound PM5-treated, VAN-treated, and CK groups are displayed in Fig. 7. With the extension of time after surgery, both of PM5-treated and VAN-treated groups showed a progressive reduction in wound area, and the scabs of the mice for both of these two group had fallen off on the day 9 as shown in Fig. 7B, whereas the CK group healed more slowly and retained a significantly larger wound area. These findings confirmed that compound PM5, which possesses antibacterial activity, exerted a marked wound-healing promotion effect. Of particular note, healing efficacy of PM5 treatment group surpassed that of the VAN group, indicating that compound PM5 may have enhanced tissue-repairing properties in addition to its antibacterial function.

Moreover, the results of HE staining revealed disorganized tissue architecture, extensive inflammation, incomplete re-epithelialization, necrosis, and vascular congestion in the CK group. VAN group showed less inflammation, partial re-epithelialization, and reduced necrosis. PM5-treated group demonstrated an almost intact epidermis, minimal inflammation, well-formed granulation tissue, and a significantly higher re-epithelialization rate compared to CK and VAN. These morphological observations were supported by Masson staining, which revealed sparse, disorganized, and weakly stained collagen fibres in the CK group, indicating impaired tissue remodelling. VAN treatment exhibited moderate, but still disorganized collagen deposition. PM5, however, exhibited dense, well-aligned, and strongly stained collagen networks, suggesting enhanced collagen synthesis and maturation. Moreover, immunohistochemical analysis confirmed these trends, with strong expression of the pro-inflammatory cytokines TNF-ɑ and IL-6 in the CK group. Their expression was reduced in the VAN group and was lowest in the PM5 group. Collectively, these results suggest that PM5 has superior healing ability for MRSA-infected wounds compared to CK and VAN by reducing inflammation, speeding re-epithelialization, and promoting collagen deposition.

## Discussion

The inexorable rise of MRSA as a leading cause of both healthcare- and community-associated infections has necessitated a continuous search for antibiotics with unprecedented mechanisms of action [36]. The COVID‑19 pandemic dramatically amplified this crisis, as widespread empirical antibiotic use accelerated the selection pressure for multidrug-resistant organisms [37]. In this context, the identification of PM5 from Lingnan medicinal resources represents a noteworthy addition to the anti-MRSA pipeline. With an MIC of 0.78 μg/mL, PM5 matches the potency of vancomycin against MRSA while displaying a broad‑spectrum Gram‑positive profile and a remarkably low tendency for resistance selection. These attributes, coupled with its membrane‑active mode of action, suggest that PM5 belongs to a growing class of compounds capable of circumventing conventional resistance mechanisms.

A distinctive feature of PM5 is its concentration‑dependent, biphasic interaction with the bacterial membrane. According to the proposed model, at sub‑inhibitory concentrations, the compound first inserts into the lipid bilayer and depolarizes the membrane, dissipating the proton motive force—a critical energy source for nutrient uptake and ATP synthesis [38]. As the concentration rises, accumulation of PM5 leads to progressive loss of membrane integrity, culminating in cell rupture and cytoplasmic leakage. This dual mechanism combines a subtle energetic disruption with a catastrophic structural failure, potentially making the bacterium less able to develop effective countermeasures. Notably, this behavior distinguishes PM5 from classical membrane‑targeting antibiotics. Daptomycin requires calcium‑dependent insertion and primarily forms oligomeric pores in the presence of phosphatidylglycerol, whereas polymyxins target lipopolysaccharide in Gram‑negative bacteria and are associated with significant nephrotoxicity [39–41]. PM5’s broad activity against Gram‑positive organisms and its apparently rapid bactericidal effect align more closely with cationic antimicrobial peptides or synthetic membrane‑active small molecules, yet it originates from a pyrrolomycin scaffold—a class more traditionally associated with halogenated natural products that exert other biochemical effects [42]. This unexpected functional repurposing highlights the potential of revisiting natural product libraries with phenotypic screening strategies.

Nevertheless, although the phenotypic analysis above established a clear mechanism of membrane activity, the depth of investigation is still insufficient. For example, it remains unclear which ion channels in the cell membrane interact with PM5 and ultimately lead to changes in the membrane potential. The electrophysiology of bacteria is crucial for the life cycle of bacteria, and Na^+^, K^+^, Ca^2+^, and Mg^2+^ plasmas have a great contribution to signal transduction [43]. Some bacteria utilize Na^+^ for ATP production or motility. K^+^ is the most abundant cation in bacteria and plays an important role in osmoregulation as well as participating in signaling within biofilms. Mg^2+^ is essential for stabilizing cell membranes and ribosomes, and Ca^2+^ is thought to be involved in cell signaling and the regulation of bacterial virulence [44]. In MRSA, changes in membrane potential are usually caused by K⁺ efflux or H⁺/Ca^2^⁺ influx, which can be triggered by membrane-active compounds inserted into the lipid bilayer or directly gated channel proteins. Although our data cannot identify the exact channel, we speculate that PM5, as a cationic amphiphilic substance, may interact with negatively charged membrane phospholipid head groups, causing local curvature stress or lipid accumulation defects that indirectly activate K⁺ -efflux systems or mechanistically sensitive channels. Alternatively, PM5 could be inserted directly into the channel pore, but this remains hypothetical.

Furthermore, in vitro antibacterial data indicate that PM5 selectively targets *S. aureus* (including MRSA) and other Gram-positive bacteria. The lack of activity against Gram-negative bacteria may be due to the permeability barrier posed by the outer membrane, which prevents many amphipathic molecules from reaching the cytoplasmic membrane. In Gram-positive bacteria, subtle variations in cell envelope components, such as teichoic acid lipidation, membrane fluidity, or surface charge, may influence PM5 binding [45]. Thus, the observed antibacterial spectrum aligns with a membrane-targeting mechanism influenced by the physicochemical properties of the bacterial envelope, rather than being dependent solely on specific receptors present only in MRSA.

It should also be noted that membrane depolarization is an early event and may not fully account for bactericidal effects. Other downstream consequences, such as disruption of ATP synthesis, mislocalization of cell division proteins, or oxidative stress, could contribute to cell death [45–47]. These possibilities warrant further investigation. In summary, although our study identifies PM5 as targeting the bacterial cell membrane, the precise molecular characteristics of the involved ion channels remain to be elucidated. Notably, the low propensity for resistance development reported for PM5 is a critical advantage. Membrane‑active agents that perturb physical integrity generally impose a high fitness cost, as bacteria would need to remodel their entire lipid composition or envelope architecture to achieve meaningful resistance [39]. Nevertheless, it is worth noting that even membrane‑active drugs can eventually face resistance: for instance, *S. aureus* can develop resistance to daptomycin through mutations that alter membrane fluidity and charge, albeit often with associated fitness defects [48,49]. For PM5, the biphasic mechanism might further elevate the barrier, because cells would need to simultaneously counteract both depolarization and membrane disruption.

From a safety perspective, the in vitro cytotoxicity data and the successful treatment of a murine skin infection model provide initial evidence of a favorable therapeutic window. Skin and soft tissue infections represent one of the most frequent manifestations of MRSA and are often amenable to topical or locally administered therapies [50]. For these indications, a potent membrane‑active agent with limited systemic exposure could circumvent the toxicity concerns that often hinder the clinical development of membrane‑targeting compounds. However, the current data do not yet clarify whether PM5’s selectivity is due to differential lipid composition between bacterial and mammalian membranes, or to the compound’s physicochemical properties (such as net charge and hydrophobicity) that favor bacterial membranes. Future work should systematically compare its hemolytic activity, mammalian cell toxicity across multiple cell lines, and, if systemic administration is envisioned, its pharmacokinetic and toxicokinetic profiles.

While the proposed membrane‑disruptive mechanism is supported by depolarization and integrity assays, the precise molecular details remain undefined. For instance, it is unclear whether PM5 acts by forming discrete ion channels, by inducing a “carpet” mechanism, or by affecting specific lipid domains [39]. Biophysical techniques such as small‑angle X‑ray scattering (SAXS), solid‑state NMR, or molecular dynamics simulations would be instrumental in clarifying the interaction at the atomic level [51]. Moreover, pyrrolomycins often possess complex halogenation patterns that influence bioactivity and metabolic stability. Systematic structure–activity relationship (SAR) studies could identify analogs with improved potency, reduced potential for off‑target effects, and more favorable pharmacokinetics [52]. The identification of PM5 as a potent anti‑MRSA agent with a novel biphasic membrane‑disrupting mechanism addresses the urgent need for antibiotics that are less susceptible to existing resistance pathways. Its efficacy in a relevant animal model and promising safety profile position it as a valuable lead for further development. Moving forward, interdisciplinary efforts combining medicinal chemistry, biophysical analysis, and comprehensive in vivo pharmacology will be essential to determine whether PM5 or its optimized derivatives can ultimately contribute to the clinical armamentarium against drug‑resistant Gram‑positive infections.

## Conclusions

In recent decades, considerable scientific efforts from both the medical community and the pharmaceutics industry have been devoted to discovering structurally diverse natural compounds against MRSA with a persistent focus on developing novel antimicrobial agents. This study demonstrated that PM5 is a selective antibacterial agent against Gram-positive bacteria, exhibiting potency similar to that of vancomycin but through a different, membrane-targeting mode of action. Preliminary investigations illustrating its antibacterial mechanism suggest that upon contact with MRSA, PM5 inserts into the bacterial cell membrane and then triggers the cell membrane depolarization. As the drug accumulates and adheres to the membrane, it directly disrupts membrane integrity upon reaching a threshold concentration, leading to membrane rupture, leakage of intracellular contents, and ultimately bacterial death. Moreover, the in vivo results towards therapeutic potential in a mouse model of skin MRSA-infection confirmed that the natural antibiotic PM5 can efficiently eliminate bacterial number, relieve the inflammation, and expedite wound healing, thereby highlighting it as a promising antibacterial candidate for the urgent need to develop novel antimicrobial agents arising from rising antibiotic resistance, particularly against MRSA. Collectively, PM5 with its high antibacterial efficacy and low toxicity, demonstrates significant therapeutic potential as a scaffold for developing next-generation anti-MRSA agents and will stimulate the creation of novel pyrrolomycin drugs.

## Data Availability

The datasets used or analyzed during the current study are available from the corresponding author on reasonable request.
